# Task-irrelevant human and robot head movements bias gaze in humans who follow them through virtual reality

**DOI:** 10.1038/s41598-026-39130-1

**Published:** 2026-02-10

**Authors:** Inka Schmitz, Jochen Miksch, Wolfgang Einhäuser

**Affiliations:** Physics of Cognition Group (212066), Chemnitz University of Technology, 09107 Chemnitz, Germany

**Keywords:** Attention, Gaze, Gaze following, Human, Robot, Virtual reality, Social interaction, Neuroscience, Psychology, Psychology

## Abstract

In prolonged social interactions, human attention and gaze are profoundly influenced by the looking behavior of others – to an extent that gaze-based communication strategies have been transferred from human-human to human-robot collaboration. Much less is known as to how gaze-based interactions play out during encounters in (semi-)public spaces, when an agent encounters another agent without them interacting towards a common goal and without the other’s gaze providing any information of relevance for the first agent’s task. Here, we investigate such a situation. We asked participants to walk behind an avatar – human or robot – who proceeded at constant speed through corridors of a virtual-reality version of a university building. Corridors had posters on the side walls. In some corridors but not in others, the avatar looked at three different posters, either 3 on the same side or 2 on one side and 1 on the other. Although the avatars’ looking behavior had no relevance for the participants, we found that participants’ gaze in a corridor was biased to the side of the posters the avatar looked at, irrespective of avatar type (human or robot). This suggests that gaze following occurs even in situations where it has no apparent benefit.

**Supplementary Information:**

The online version contains supplementary material available at 10.1038/s41598-026-39130-1.

## Introduction

Human gaze is guided by a variety of factors. These may arise from the stimulus – ranging from low-level “salience”^1,2^ across mid- and high-level features^3,4^ to proto-objects^5^ and objects^6^ – from the current goals and tasks^7–10^ or represent individual traits^11^. When acting in the real world, additional constraints impact gaze behavior. This includes, for example, maintaining safe gait and planning the path when negotiating terrain^12–14^ and the active compartmentalization of complex tasks, such as tea or sandwich making^15,16^, into individual steps^17^. The effect of such constraints on gaze can be substantially modulated by instruction; for example, mentioning stairs substantially increases gaze to the stairs compared to an otherwise identical task^18^.

In social settings, the gaze of others plays a highly important role in guiding an individual’s own gaze. Even in abstracted form such as a cartoon face, gaze direction presents a strong attentional cue^19,20^, which is hard to ignore^21^. Such automaticity or reflexivity of gaze following has been argued to reflect a fundamental mechanism of social attention rather than a strategic use of information^22^. Understanding and expressing gaze behavior is essential for the smooth interaction between humans^23^. Even young children show the ability to follow gaze, interpret gaze and to signal their own intentions and direction of attention to others^24^. Adult humans can reliably estimate the gaze direction of others^25^, although some misattribution may happen if head and eye direction are not aligned, where the head direction dominates the estimate^26,27^. A meta-analysis by McKay and colleagues^28^ further confirms that gaze-cueing effects are robust across a wide range of experimental settings and persist even when gaze cues are task-irrelevant, but there is less research in more complex or realistic settings. Investigating gaze following under naturalistic conditions is, however, needed to disentangle automatic social attentional biases from goal-directed or instrumental uses of gaze information. The relevance of gaze interactions is not restricted to the human-human case but increasingly used in the design of artificial agents to provide them with human-like interaction capabilities^29^. Robot gaze cues can drive human attention even without visible eyes, indicating that artificial agents are capable of eliciting social attentional processes similar to human agents^30^. However, these effects can vary depending on agent morphology, context, and task reliability^29,31^, and can even interact with participants’ social-affective traits such as anxiety or threat sensitivity^32^. Therefore, agent type (human vs. robot) may modulate attentional and social responses, making this a relevant example for studying gaze-following in complex but controlled settings. Moreover, also within the same agent type and even within the same agent, their appearance and movement dynamics may influence interaction behavior. Hence, we present the agents as avatars in a naturalistic virtual-reality setting, which ensures their consistent appearance and dynamics. Moreover, we chose the avatars’ appearance as neutral as possible and chose a standard (smooth and non-exaggerated) walking style for the human and had the robot “slide” (i.e., moving smoothly as if moving on wheels and tracks) forward.

Most studies on gaze in naturalistic human-human and human-robot interaction are concerned with situations in which the agents interact with each other for a prolonged time or follow a common goal, for which they require shared or joint attention to at least some degree^33–35^. In the real-world, however, humans often interchange gaze information with others during subsequent brief, incidental encounters with a large set of different individuals – think of making your way through a crowded train station as compared to having a conversation at the dinner Table^36^. This distinction is critical, as humans adapt their gaze behavior during brief encounters to the (in)action of the other party^37^. Moreover, when a visually conspicuous item is placed in a busy corridor, passersby tend to follow the gaze of people walking in the same direction more than of people approaching them, suggesting that the layout of the encounter plays a further crucial role for mutual gaze guidance^38^.

The two aforementioned studies^37,38^ pioneered studying gaze in incidental encounters with two complementary approaches. While Hessels and colleagues^37^ relied on a substantial number of confederates to induce different kinds of encounters with the participants, Gallup and colleagues^38^ relied on truly incidental passersby, which required the authors to place a conspicuous, out-of-context item to gather sufficient data. Provided the evidence that gaze cues can elicit automatic attentional shifts even when they are task-irrelevant^22,28^, we here asked whether gaze following also occurs in a dynamic, naturalistic setting when the gaze of the other individual is neither informative nor beneficial for the participant’s instructed task and when no specifically conspicuous item is present. Hence, we could follow neither of the previous approaches. Instead, we used virtual reality (VR), which allowed us to balance the needed degree of experimental control with the naturalism of the scenario, without the need for well-trained confederates. Moreover, it allowed us to study not only human-human gaze following, but also to replace the human by a robot who showed virtually identical behavior. Specifically, we instructed participants to follow a human or a robot avatar through a series of corridors. The avatar intermittently glanced at posters at the corridors’ sidewalls. This approach allowed us to examine basic gaze-following under clean, well-controlled conditions while maintaining naturalistic task engagement. We had hypothesized that the avatar’s gaze behavior, despite bearing no information for the participant’s task, nonetheless biased the participant’s own gaze. In addition, we explored whether the type of avatar – human or robot – impacted these results.

### Results

**Fig. 1:**
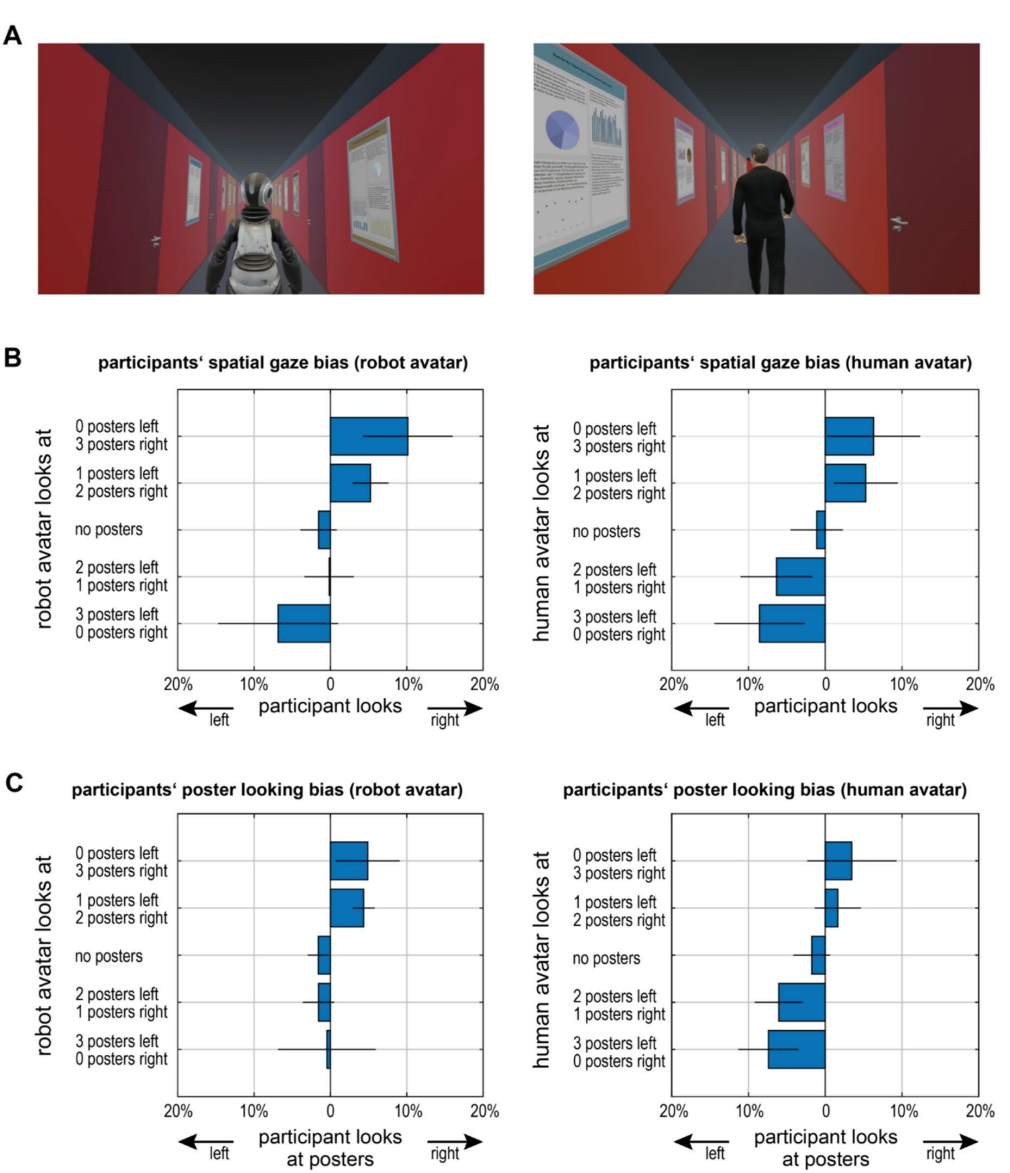
(**A**) Example frames from the perspective of the participant, following a robot avatar who glided smoothly (left) or a human avatar who walked (right) through the corridor. A movie with examples of different conditions and superimposed gaze is available as SI Video 1 and at 10.17605/OSF.IO/UPKQ6. **(B**) Participant *spatial gaze bias* (defined as fraction of time looking to the right minus fraction of time looking to the right, with left/right defined as exceeding 5 degrees of visual angle around the center) as function of avatar looking behavior for the two avatar types (*left*: robot, *right*: human). (**C**) Participants’ *poster looking bias* as function of avatar looking behavior. In contrast to panel B only participants’ looks at posters are counted as looks to the left or the right respectively; otherwise, notation identical to panel B.

We asked 16 participants to follow an avatar – human or robot – through a series of virtual corridors (Fig. 1A) in a within-subject design. Participants controlled their forward speed and lateral position, but a speed restriction ensured that they could not get closer than 2 m to the avatars and therefore could not overtake them. On the side walls of each corridor there were ten posters on each side, whose layout resembled those typically seen in scientific contexts (see Methods – VR Environment for details). No additional instruction or task was given other than to walk behind the avatar; in particular, there was no reference to avatar gaze or posters. The corridors differed in the avatar’s looking behavior: In half of the corridors, the avatar did not look at any posters. In the other half, the avatar glanced at three posters, which could either all be on the same side (12.5% of all corridors) or two posters on one side and one poster on the other (37.5% of all corridors). That is, we had five different levels of *avatar looking behavior* (sorted by increasing avatar looking bias to the right: 3 posters looked at on the left/0 on the right; 2 posters on the left/1 on the right; 0 on either side, 1 on the left/2 on the right, 3 on the right/0 on the left). The order of avatars, the order of corridors with and without the avatar looking at posters as well as the general layout used (determining turning directions) were balanced in a way to minimize any potential order effects (see Methods – Procedure for details). To determine whether the factors *avatar looking behavior* and *avatar type* influenced the participants’ gaze, we for each of the 10 combinations (5 avatar behaviors × 2 avatar types) subtracted the fraction of time a participant looked more than five degrees to the left from the fraction of time the participant looked more than five degrees to the right. We found this measure of participant *spatial gaze bias* to be influenced by the avatar behavior (F(4,60) = 3.98, *p* = .006, repeated-measures analysis of variance [rmANOVA]). When the avatar looked at more posters on the left, the participant’s gaze was biased towards the left; when the avatar looked at more posters on the right, the participant looked more to the right (Fig. 1B; Table 1). We did not observe any effect of *avatar type* (human/robot, F(1,60) = 0.31, *p* = .587) nor an interaction between *avatar looking behavior* and *avatar type* (F(4,60) = 0.20, *p* = .936). This shows that the gaze of the avatar yields gaze following behavior independent of whether the avatar is a human or a robot.

**Table 1:** Mean and standard errors of mean (in parenthesis) for the *Spatial gaze bias* and *poster looking bias* as function of *avatar looking behavior* (rows) and *avatar type* (columns).

Posters looked at by avatar (right/left)	Spatial gaze bias		Poster looking bias	
	Robot	Human	Robot	Human
3/0	+ 10.1% (5.9%)	+ 6.3% (6.1%)	+ 4.9% (4.2%)	+ 3.5% (5.8%)
2/1	+ 5.2% (2.3%)	+ 5.3% (4.2%)	+ 4.3% (1.4%)	+ 1.6% (3.0%)
0/0	− 1.6% (2.4%)	− 1.1% (3.4%)	−1.6% (1.4%)	−1.8% (2.4%)
1/2	− 0.2% (3.2%)	− 6.3% (4.7%)	−1.6% (2.0%)	−6.1% (3.1%)
0/3	− 6.9% (7.9%)	− 8.6% (5.9%)	−0.5% (6.4%)	−7.4% (3.9%)

Our first analysis only considered whether the participants’ gaze pointed to the left or right, irrespective of whether they actually looked at a poster. Indeed, there is considerable inter-individual variability in the fraction of time spent looking at posters, which ranges from 1.0% to 53.4% of time (mean: 19.1%, sd: 15.6%). This time did not depend on *avatar type* (F(1,60) = 3.00, *p* = .103) nor on *avatar looking behavior* (F(4,60) = 1.39, *p* = .247). Neither was there an interaction between these factors (F(4,60) = 2.26, *p* = .073). Nonetheless, the difference between the fraction of the participant looking at posters to the right minus the fraction looking at posters to the left, the *poster looking bias* (see Methods – Analysis – Measures for details) showed a similar qualitative pattern as the general participant looking bias (Fig. 1C; Table 1), though this less robust measure failed to reach significance (both main effects and interaction: *p* > .136). In sum, our data show that participants’ general looking direction is robustly influenced by the avatar looking at the posters on the side walls, though this bias is not necessarily poster-specific.

### Discussion

We demonstrate that the gaze direction of an avatar – human or robot – biases the gaze behavior of participants walking behind them in a naturalistic virtual environment. Importantly, this gaze following occurs, although neither the avatars’ gaze dynamics nor their gaze targets convey any information of relevance for the participant’s task.

Our results corroborate earlier findings that gaze following is a robust mechanism, even without task relevance or explicit social interactions^38^. In contrast to Gallup and colleagues, our setting did not require any specific conspicuous items. In our case, the objects (posters) were deliberately designed that they were neutral regarding social context (it was neither inappropriate to look at them nor to ignore them), about equal in visual salience without being too conspicuous, not task-relevant and fitting in the context of the environment (the actual building the corridors were modelled after, has scientific posters on some walls). Moreover, the sides of the corridors were balanced to each other – they had the same structure, the same number of posters, the participants started centrally in the corridor and directional information for the upcoming turn became only available on the final stretch and did not relate to the preceding looking behavior of the avatars. Hence, it is solely the looking behavior of the avatar that is responsible for the gaze bias in the participant. It would, however, be straightforward to also test such gaze following behavior in more complex environments with multiple avatars and the need to avoid collisions^39^ or objects that are not neutral in one or more respects – for example, highly salient posters, posters that are in themselves interesting or inappropriate to look at, or other objects that contain task-relevant information or do not fit the environment^40^. While clearly beyond the scope of the present study, this would allow assessing how much incidental gaze following remains, if other gaze attracting (or repelling) factors are available.

Despite the robust gaze bias towards the *side* the avatar preferentially looked at, we did not find a bias towards the specific objects (posters) the avatars looked at. While we cannot exclude that this measure is somewhat more brittle – in fact, the overall bias pattern (Fig. 1C) looks qualitatively similar to the pattern for the general direction (Fig. 1B), but does not reach statistical significance – it also may reflect that *object-related* gaze following becomes weaker in the absence of social interactions. Real-life gaze behavior in incidental encounters is clearly modulated by the degree of social interaction sought by the observer’s counterpart^37^. Hence, it seems plausible that if avatars were to actively engage with the participants, participants’ looking behavior would tie more closely to the avatars’ actual gaze targets. Moreover, the avatars’ gaze may direct participants’ attention to the poster in general, but not all of the participants’ attention shifting needs to be overtly expressed. It is also conceivable that the precise gaze targets of the avatars are not clearly evident to the participant from the avatars’ head rotation, whereas the overall looking direction (left or right) clearly is. In any case, participants’ gaze following towards the side preferentially looked at by the avatar is robust, irrespective of their gaze’s precise target.

The complete irrelevance of the avatar’s gaze behavior for the participant constitutes a critical aspect of the present design. This is in stark contrast to typical studies of gaze following with humans or robots, which often focus on using gaze-based interactions to effectively and efficiently complete a task jointly, and using gaze to establish joint or shared attention^33,34^. It should be noted, however, that while the avatars’ *gaze* is irrelevant to the task, the avatars’ motion *as such* is not: participants are instructed to “follow” them, which may imply keeping a comfortable distance (only the minimum distance was constrained) and the avatar needs to be observed to make the correct turn direction at the corridors’ ends. Consequently, there is some form of “social” engagement with the avatar, which might encourage gaze following, even if it is neither required nor beneficial.

We deliberately chose to avoid any specific instruction other than following the avatar. The actual instruction (in German) was also chosen such that there was no possible misconception of “following” in a metaphoric sense (like in “follow the example of”), but the phrasing instead only referred to the spatial act of walking behind the avatar (specifically, the word “*folgen”* [to follow] was avoided, instead “*hinterher laufen*” [approx. to walk behind] was used). We consider this critical, as even in non-social situations, instructions can have a profound impact on gaze^18^. It is likely that each participant interpreted the task somewhat differently and the fact that there are large interindividual differences in the time spent looking at posters supports this assumption. However, this makes it even more striking to observe a consistent and robust spatial bias induced by the avatar, irrespective of the individual’s preference for exploring the space or the posters.

In our study, participants saw the avatars exclusively from behind, such that gaze information is available only from head movements. This exclusion of eye-based cues may present a limitation to the ecological validity of the study and is an extension to be addressed in future research. For the present purpose, the restriction to seeing only head movements allows us to clearly separate the roles of the avatar as sender of gaze information from the participant as receiver thereof. This prevents participants from assuming a two-way interaction and from forming an impression about the (in)adequacy of the avatars’ responsiveness^23^, which would likely influence them in complex ways for human and robot avatars^41^. Moreover, in the absence of any benefit of gaze interaction, gaze following can be stronger when encountering others from behind in a corridor than when approaching them face on^38^ but different to this field study, contrary effects have been shown in the lab^42^. Hence, observing gaze following in the absence of a specific task is somewhat limited in scope, but our setting could be readily extended to test how the observers’ task modulates their gaze following. A further experimental challenge in head-on encounters is given by the fact that realistic encounters than would imply a variety of interaction partners (avatars), such that interindividual differences in the contribution of eye and head to gaze adjustment^43,44^ are likely of relevance for the avatar model. Hence, while head-on encounters are clearly a most interesting issue for further research, they are clearly beyond the scope of the present study.

The sample size in our study (*N* = 16) was relatively small, though sufficient to detect large effects in a within-subject design. Indeed, we found the hypothesized effect of the avatars’ gaze influencing observers’ gaze behavior, which suggests that the effect is robust and of relevant size. Nonetheless, it is conceivable that we may have missed smaller effects; for example, subtle differences between the avatars, complex interactions between gaze and avatar type, or significant poster looking biases. Another limitation is the representativeness of the sample, which was comprised mostly of young adults undergoing university education. As looking behavior in VR is modulated by age^40^ and the perception of robots depend on age and education^45^, our results, in particular for the robot avatar, may differ in samples that cover the human life span more evenly. For the specific sample, it is also possible that the participants knew part of the texts presented on the posters, which were designed to be plausible in the context. However, we consider it highly unlikely that this affected our data – especially, as participants very rarely stopped or engaged in extensive reading behavior. A further potential limitation concerns the avatars themselves: both moved at a constant speed, the human avatar displayed a walking animation while the robot glided without limb articulation, and neither responded dynamically to participant movements, which may have influenced gaze following. Moreover, only one appearance was chosen per avatar type, both the human and the robot were selected to appear neutral, neither threatening or authoritative nor cute or submissive. The interaction with distinct “individuals” as avatars may yield to more behaviorally variability in the participants, especially if the individuals exhibit different appearance and/or dynamics. Finally, the controlled VR environment with a simplified corridor layout, uniform poster presentation and a smooth movement control, while useful for experimental control, may not fully capture the attentional demands of real-world navigation and locomotion. Given our results, studies on gaze following that manipulate the avatars appearance, dynamics and interaction behavior as well as use head-on encounters with the avatars’ eyes visible, will be interesting extensions of the present work, combining naturalistic settings with a high degree of experimental control. The fact that systematic effects were observed even when gaze was task-irrelevant and avatars were seen only from behind highlights the robustness of these social attentional mechanisms and opens a promising avenue for this line of research.

When eye and head are free to move, gaze in virtual reality presents a good approximation of gaze in the real world^46^. Besides the good experimental control, studying gaze following in a virtual reality has the advantage that avatars can be used for which and whom no real-world counterpart exists. For human avatars, this allows controlling appearance, gender, familiarity, gait patterns, size, speed, etc., in a way impossible to achieve with human confederates. For robots, it allows exploration of these features without the necessity to build the actual robot. Since social gaze in human-robot interaction may depend on robot appearance^29^ and gaze following might in turn be indicative of more abstract constructs like joint and shared attention^33,34^, using distinct robot avatars and assessing their effect of gaze following might allow unobtrusive studies on these constructs in naturalistic settings. The ever-growing prevalence of embodied digital technologies in public spaces, which includes autonomous agents like delivery robots or autonomous vehicles, will increase the necessity of smooth human-machine interaction in incidental public encounters. Our observation that in a simple setting – where gaze following is neither task-relevant nor beneficial – humans are biased by the head rotation of a robot similar to the head rotation of a conspecific presents one further step in making the usefulness of (simulated) gaze to facilitate such interactions likely.

## Methods

### Participants

Sixteen volunteers (13 women, 3 men, age: 19–28 years; mean: 21.4 years; sd: 2.7 years) participated in the study. The sample size of 16 had been chosen, as it would allow detecting large effects (f ≥ 0.40) with a statistical power of 1 − β = 0.80 at an alpha level of 0.05 for any two-level comparison. All participants had normal or corrected-to-normal vision and participants provided written informed consent prior to participation. Participants were compensated with either course credit or €10/hour. All procedures were performed in accordance with the tenets of the Declaration of Helsinki and in accordance with all applicable guidelines and regulations. All procedures were approved by the local ethics review board (*Ethikkommission der Technischen Universität Chemnitz*, case no. #101607178).

### Apparatus

Experiments were conducted in a laboratory setting with controlled environmental conditions. The virtual environment was rendered using an HTC Vive Pro Eye headset (dual OLED screens, 2880 × 1600 pixels resolution, 90 Hz refresh rate), offering a maximum field of view of 110 degrees. The system was powered by a Bestware XMG NEO 17 laptop (AMD Ryzen 9 5900HX CPU, 32 GB RAM, Nvidia RTX 3080 Mobile GPU). Eye-tracking data was recorded at 120 Hz by the system built-in in the head-mounted display.

### VR environment

The virtual reality, in particular the corridors’ layout, size (width: 2.4 m, length: 27 m) and color scheme, was based on previous studies^46,47^, which in turn had used realistic models of the university building the authors’ offices and labs are located in. Each corridor featured ten posters on either side, designed to resemble typical scientific posters. These posters were randomly generated using JavaScript, incorporating text from university news pages, along with randomly created graphic elements, layouts, and color schemes to ensure diversity in appearance but consistency in style (Fig. 1A). Posters were of size ISO A0 portrait (0.84 m wide, 1.2 m high), their center was 1.6 m above the floor. On each side there were also 3 closed doors, randomly placed in lieu of a poster. That is, there were a total of 13 locations on each side, three of which were occupied by doors, the other ten by a poster.

The avatars were implemented in Unity. We started with a free rigged character from MakeHuman^48^ and a robot (Cute Robot 3) from the Unity Asset Store^49^. Both models were exported to Blender, where the meshes were cleaned and the armatures adjusted, before being re-imported into Unity as humanoid rigs. Avatars moved at a constant speed of 0.9 m/s along the center of the corridor; while the human avatar exhibited an animated walking movement, the robot avatar glided in front of the participant without any limb articulation, but expressed only head rotation.

We transferred the participant’s actual head orientation directly to VR. Since naturalistic gaze orientation, which includes head and eye movements^43^, is crucial to our study, we refrained from using the physical head orientation for any other control, although such strategies are frequently employed in other VR contexts^50^. When in the corridors, participants’ longitudinal and lateral movements were smooth (i.e., no teleportation was used or possible) and they could adjust their velocity smoothly. To avoid distractions from following the avatar, we used a comparably simple movement control for lateral position and longitudinal speed: With a handheld controller, participants could continuously adjust their speed within a defined range (0.6–1.2 m/s). To avoid overtaking the avatar, participants could not get closer than 2 m to the avatar (distance to avatars’ center); whenever participants were about to exceed this limit, their speed was reduced to the avatar’s speed of 0.9 m/s. With the same controller, participants could adjust their lateral position, but not get closer than 0.3 m to either wall. Participants reported no difficulty in using the movement controls. On average, they held a distance of 3.0 m (sd: 0.92 m) from the avatar; this distance did not depend on avatar type or avatar looking behavior nor was there an interaction (all *p* > .296).

At the end of each corridor, there was a T-intersection at which the avatar turned either left or right. Participants were required to move slightly laterally to continue following the avatar by aligning themselves with an appropriate lane. The information in which direction to turn was available only once the avatar started their turn at 2.5 m distance from the end of corridor, ensuring that the turning process did not interfere with the looking behavior at posters, as no poster was closer than 5 m to end of the corridor. To avoid potential simulator sickness by the turning event, participants were “teleported” around the corner as soon as they reached the end of the corridor. After turning correctly, the participants found themselves at least 2 m behind the avatar in the new corridor. In the rare cases, in which participants turned in the wrong direction (3 participants made this error once, all others never), they were teleported to a dead-end corridor, where they had to wait for 5 s before they were teleported to the correct corridor.

### Procedure

Each participant completed a total of 32 corridors, over the course of four experimental blocks of eight corridors each. In 50% of the corridors (4 per block), the avatar ignored the posters, while in the other 50%, it glanced at three posters. These glances were either directed at all posters on one side (12.5% of corridors, 1 per block) or distributed between both sides (37.5%, 3 per block). For the latter case, all possible orders (left-left-right, left-right-left, right-left-left and right-right-left, right-left-right, left-right-right) were used exactly once per participant and avatar type. Each participant completed two experimental blocks with a human avatar and two with a robot avatar. At the start of the first corridor of each block, participants were placed 3 m behind the avatar. The order of experimental blocks was either human-robot-robot-human or robot-human-human-robot.

For each block, there were two possible orders of corridors in which avatars looked (L) at posters and of those in which they did not (N), either LNNLNLLN or NLLNLNNL (i.e., mirrored versions of each other), each of which was used once per avatar type. The eight corridors were arranged into two labyrinth designs, with equal numbers of left and right turns. Mirrored versions of the labyrinths were also created, resulting in four possible configurations. The 16 different combinations of avatar order (2), corridor-looking order (2) and corridor configuration (4) were counterbalanced across observers (i.e., each combination was used for exactly one participant).

To familiarize participants with the controls and the task, prior to the first experimental block, a block of four shorter corridors, in which the avatar did not look at any poster was conducted. The avatar type in this training block matched the avatar of the first experimental block.

The eye tracker was calibrated before each experimental block with the device’s internal procedure. When participants reached the end of the final (8th ) corridor in a block, they were teleported to a nineth corridor without an avatar, where a custom validation procedure of the eye tracker’s calibration was performed. To this end, five dots were presented in front of the participants. For one of the participants, the recording of the validation procedure failed due to a technical error, in the other 15 we found that error was mostly due to drift (i.e., a shift of the entire pattern rather than scaling or rotation), with an average error of 3.6° (sd: 1.7°) in the horizontal and 4.5° (sd: 2.4°) in the vertical. Importantly there was no bias to either side (average signed horizontal error: 0.05° (sd: 3.6°)). Together with the fact that each avatar looking behavior was shown in each block, this rules out that measurement errors could cause the observed pattern of results. Since we have no reason to assume that measurement error was worse in the participant for whom validation recording failed, we included all 16 in the analysis.

The experiment’s net duration ranged from 18.3 min to 20.6 min (mean: 18.5 min, sd: 0.54 min).

### Data analysis

#### Recording

Raw data was recorded within Unity 2019.4, consisting of eye-tracking data and positional headset data. From these data viewing angles (“gaze in world”) were calculated and gaze “hit points” (i.e., the first intersection of the line of sight with an object) utilizing colliders for the avatars, hallways and posters. Since the phase of the human avatar’s walking animation was not synchronized with the presentation, there is some minor uncertainty about the area actually covered by the avatars’ body parts. For the analysis using the poster hit points, we for each frame computed the volume the respective body part could possibly occupy (i.e., a volume slightly larger than the volume actually occupied). For comparison, we also computed the volume the respective body part certainly occupies (i.e., a volume slightly smaller than the volume actually occupied), and the differences were negligible.

#### Pre-processing and data exclusion

All analyzed data were extracted frame-wise by Unity 2019. The raw coordinates are absolute in the general layout (world) relative to a fixed Cartesian coordinate frame (x, y,z). Angles for head and gaze direction were re-referenced to the direction of the corridor (0° is the forward direction, negative angles to the left, positive angles to the right). In particular, gaze directions were transformed into this coordinate frame by combining eye-in-head direction (origin centered on the forehead, corresponding to a cyclopic eye) and head-in-world direction (which is available in word coordinates). No other transformations were applied. Collisions of the gaze vector with avatars, hallway components (wall, ceiling, doors, etc.) and posters were taking from Unity and verified to match the raw location and direction data. Periods in which the eyes were closed or data were unavailable (3.4% of total time) were excluded from all analyses, all other samples constitute the valid data used for analysis.

#### Measures

From the valid data, we computed the following measures that entered statistical analysis as dependent variables:

- *Spatial Gaze Bias* (Fig. 1B): For each participant and condition, the time spent looking more than 5° to the left and right of the central line of sight was summed. Gaze bias was computed by subtracting right from left values and dividing by the total valid time. Since the 5° threshold is somewhat arbitrary, we recomputed the measure using a 10° threshold and found that this did not alter the pattern of results.
- *Poster Looking Bias* (Fig. 1C): The time that gaze hit points fell on posters were summed separately for posters on the left and the right wall; the sums were subtracted from each other and divided by total valid time.

The following measures or experimental manipulations were used as independent variables for the statistical analysis (factors in the rmANOVA):

- *Avatar Type* with 2 levels: Human, Robot.
- *Avatar Looking Behavior* with 5 levels: 3L0R, 2L1R, 0L0R, 1L2R, 0L3R, where nL means the avatar looks at n posters at the right wall, and mR that the avatar looks at m posters at the right wall.

The corridor layouts and thereby the turn directions were counterbalanced across observers, but do not enter the analysis. Similarly, the presentation order of levels in the factors was designed to prevent any order effects (see Procedure for details).

Data was processed using Matlab (The MathWorks Inc, Natick, MA), statistical analysis were conducted with R^51^.

## Supplementary Information

Below is the link to the electronic supplementary material.

Supplementary Material 1

Supplementary Material 2

## Data Availability

All data needed to replicate our analyses are available along with example scripts at https:/doi.org/10.17605/OSF.IO/UPKQ6.
