# Supplementary material for: Task-irrelevant human and robot head movements bias gaze in humans who follow them through virtual reality
Source: Sci Rep. 2026 Feb 10;16:5563. doi: 10.1038/s41598-026-39130-1 (PMC12891715; doi:10.1038/s41598-026-39130-1)
Supplement: Supplementary file 1 — Supplementary Material 1 [file 41598_2026_39130_MOESM1_ESM.pdf]

Supplementary Information

Supplementary Video Legend

SI Video 1. Illustration of VR Environment. A video of the avatars from a participant's perspective with gaze data (blue dot indicates gaze "hit point") overlaid. The sequence includes two corridors with the human avatar, followed by two corridors with the robot avatar; for each avatar type there is one corridor with gaze to the poster, one without. The light gray on the floor towards the end of the corridor indicates the participant's current turning choice (by their lateral position), not the subsequent turning of the avatar, which becomes only evident at the very end of the corridor. A full resolution video is available together with the data at https://doi.org/10.17605/OSF.IO/UPKQ6.
